# Supplementary material for: High gamma response tracks different syntactic structures in homophonous phrases
Source: Sci Rep. 2020 May 5;10:7537. doi: 10.1038/s41598-020-64375-9 (PMC7200802; doi:10.1038/s41598-020-64375-9)
Supplement: Supplementary file 1 — Supplementary Information. [file 41598_2020_64375_MOESM1_ESM.pdf]

High gamma response tracks different syntactic structures in homophonous phrases

Fiorenzo Artoni[1,2], Piergiorgio d'Orio[3,4], Eleonora Catricalà[5], Francesca Conca[5], Franco Bottoni[6], Veronica Pelliccia[3,4], Ivana Sartori[3], Giorgio Lo Russo[3], Stefano F. Cappa[5,7*], Silvestro Micera[1,2*], Andrea Moro[5]

Affiliations

[1]The Biorobotics Institute, Scuola Superiore Sant'Anna, Pisa, Italy.

[2]Translational Neural Engineering Laboratory, Center for Neuroprosthetics and Institute of Bioengineering, EPFL – Campus Biotech, Geneve, Switzerland.

[3] "Claudio Munari" Center for Epilepsy Surgery, ASST Grande Ospedale Metropolitano Niguarda, Milan, Italy

[4] Institute of Neuroscience, CNR, Parma, Italy

[5] Neurocognition Epistemology and theoretical Syntax Research Center (NEtS), Scuola Universitaria Superiore IUSS, Pavia, Italy.

[6] Istituto Clinico Humanitas, IRCCS, Rozzano, Italy.

[7] IRCCS Mondino Foundation National Institute of Neurology, Pavia, Italy

Co-corresponding authors

SM (Silvestro.micera@epfl.ch[santannapisa.it]), AM (andrea.moro@iusspavia.it)

Supplemental Data Items

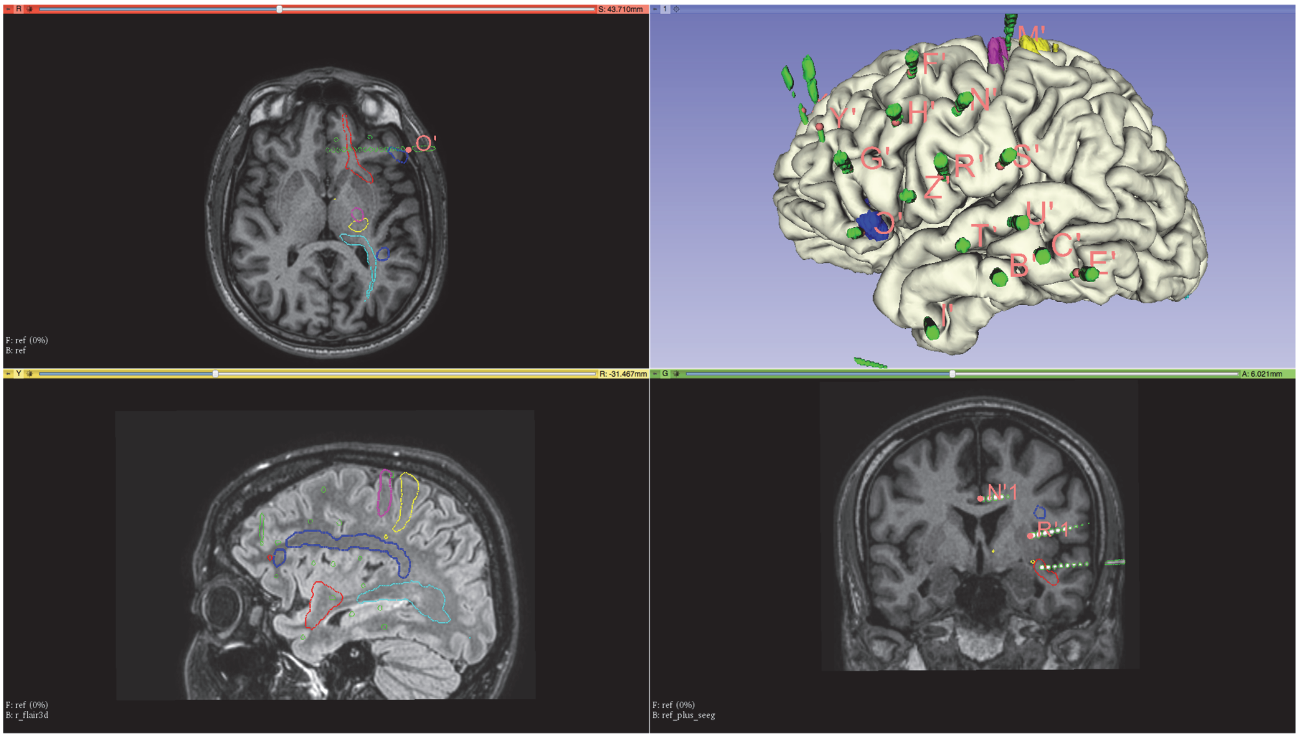

Ext. Data Figure 1: Example of Multimodal and interactive scene assembled with 3D Slicer software of patient s11.

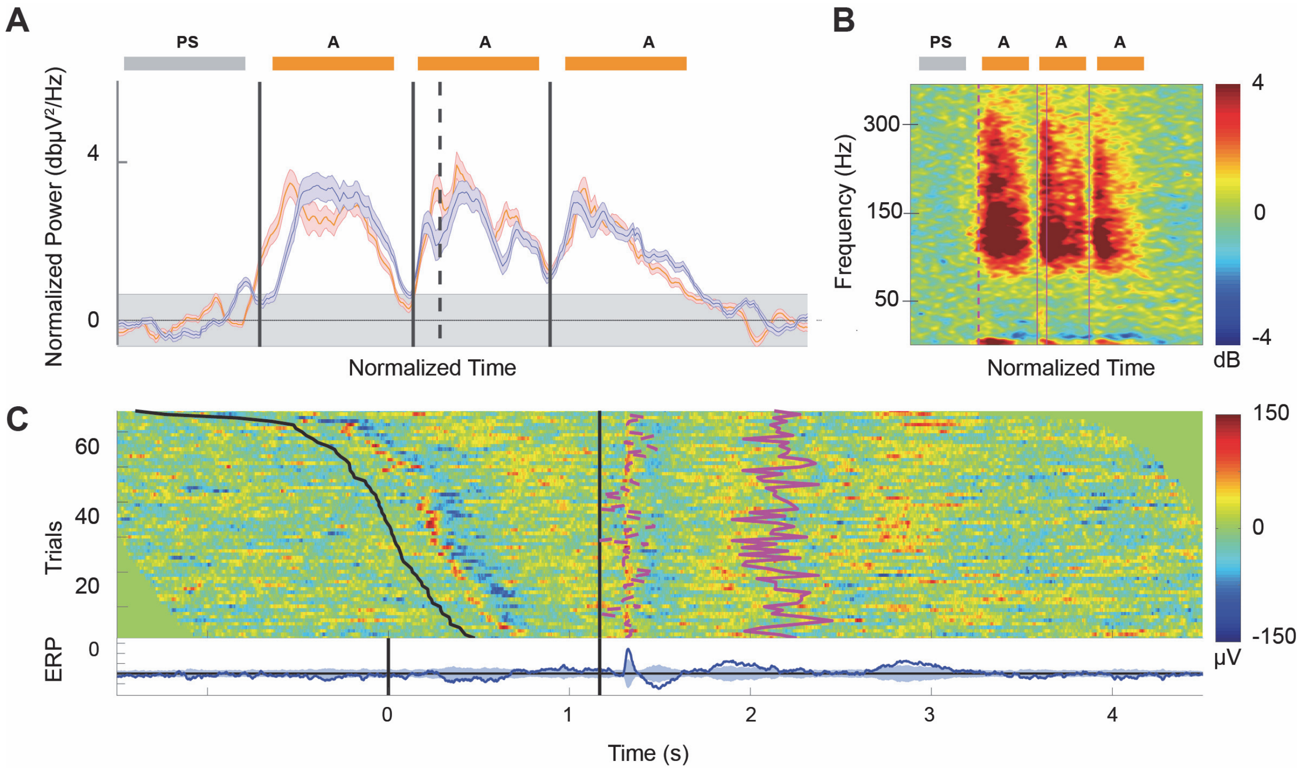

Ext. Data Figure 2: Heschl ERPImage and Time Frequency. A) Band-specific (150 -300 Hz) ERSP (bERSP) for VPs (red) and NPs (blue) respectively for a Heschl contact. The four vertical bars separate the auditory stimuli (A) from the prestimulus interval (PS) and respectively indicate the beginning of the phrase, the beginning of the Art/Cl, the beginning of the word immediately following Art/Cl (Verb/Noun), the beginning of the word after. B) Time-warped Event-Related Spectral Perturbation (ERSP) of verb and noun phrases pooled together. The four vertical bars have the same meaning as in the baseline-normalized power plots (panel A). C) Event-related single-trial potential image (ERPImage) time-locked to the stimulus presentation. Trials are aligned to the beginning of Art/Cl (continuous black vertical line). The first black line indicates the beginning of the phrase for each trial. The following two pink lines respectively indicate the beginning of the word immediately following Art/Cl (Verb/Noun) and the beginning of the word after, for each trial.

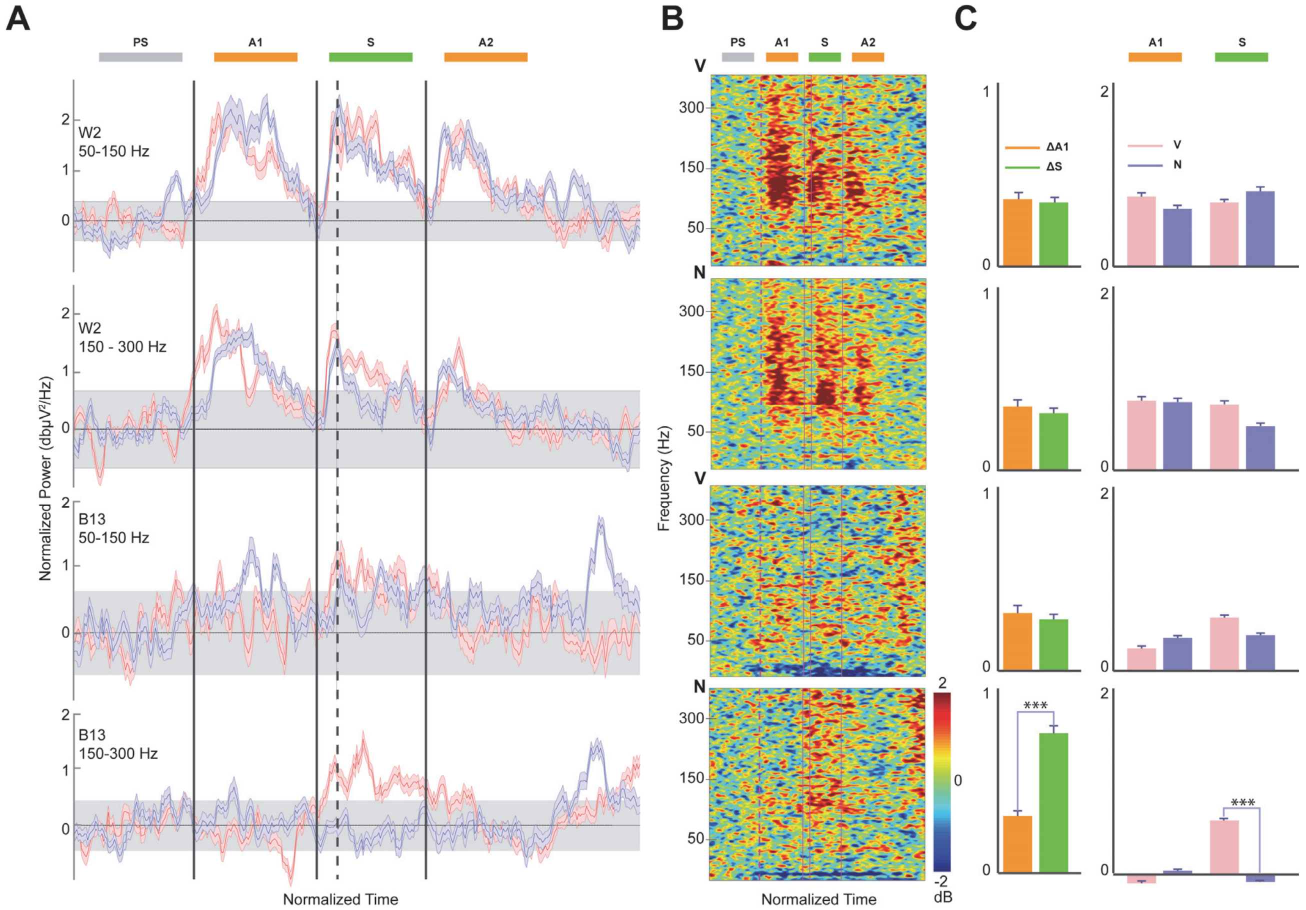

Ext. Data Figure 3. Band-specific ERSP relative to the [50 - 150] Hz and [150 - 300] Hz frequency intervals for a responsive contact (B13) and a non-responsive contact (Heschl - nonspecific auditory response). VPs are represented in pink, NPs in blue. The four vertical bars separate the regions pertaining to the auditory stimuli (A1, S, A2) from those within the pre-stimulus interval (PS). They respectively indicate the beginning of the phrase, the beginning of the Art/Cl, the beginning of the first word immediately following Art/Cl (Verb/Noun), the beginning of the word after. A1 and A2 refer to the auditory stimuli respectively before and after the tROI (Art/Cl + N/V syntax construct). B) Time-warped Event-Related Spectral Perturbation (ERSP) respectively for VPs and NPs. The four vertical bars and indicators have the same meaning as in the baseline-normalized power plots (panel A). C) From left to right the bars represent (i) the absolute value of the normalized power difference between VPs and NPs in the intervals A1 (orange) and S (green), (ii) the absolute value of the normalized power of VPs (pink) and NPs (blue) in the intervals A1 and S respectively.*** = $p<0.001$

ID	Tot implanted contacts (tot implanted electrodes)		RC num	SITE OF SC lead number (electrodes num)																													
				Orbital cortex		Superior frontal gyrus (areas 8-9)		SMA (area 6)		Middle frontal gyrus		Inferior frontal gyrus (areas 44-45)		Frontal cingulate gyrus (area 24)		Fronto-parietal operculum		Insular cortex		Temporal pole		Superior temporal gyrus		Middle temporal gyrus		Inferior temporal gyrus		Temporo-basal cortex		Temporo-Occipital cortex (area 19)		Inferior parietal lobule	
	DH	NDH		DH	NDH	DH	NDH	DH	NDH	DH	NDH	DH	NDH	DH	NDH	DH	NDH	DH	NDH	DH	NDH	DH	NDH	DH	NDH	DH	NDH	DH	NDH	DH	NDH	DH	NDH
s03	0	264 (19)	59				1				1		5		13(3)		3		6(3)		2				11 (3)		6 (2)		9(2)		2		
s06	168 (13)	0	18	1								1		1		2		1						8 (2)		2		2					
s09	0	272 (19)	11		1						2		1		4(2)														2				1
s10	225 (16)	0	71											1						5		6(2)		13 (3)		10 (2)		5		12 (3)		13(3)	
s11	219 (17)	0	18					1		2		2				3(2)								8 (2)		2							
s12	0	210 (15)	2		1										1																		
s13	210 (18)	0	6			1												4(2)				1											
s17	0	206 (15)	5																						3								2
s21	0	205 (15)	43																3		8		2		12 (2)		5 (2)		11(3)		2		
s23	25 (3)	200 (14)	9										4(2)				4																1

Ext. Data Table 1: Responsive contacts. Highlights of responsive contacts in the whole population of patients that completed the study. RC: Responsive Contacts; SMA: Supplementary Motor Area; DH: Dominant Hemisphere; NDH: Non-Dominant Hemisphere. 6 leads (3 electrodes) in patient s10 showed an auditory response into the mesial cortex of occipital lobe.

	Noun-phrase				Verb-phrase			
	Article				Pronoun			
	valid	*miss %*	*mean*	*std*	*valid*	*miss %*	*mean*	*std*
freq. Repubblica form	30	3.23	1.45	0.50	21	32.26	2.95	0.52
freq. ITWAC form	31	0	1.46	0.48	22	29.03	2.84	0.64
freq. Repubblica lemma	30	3.23	1.14	0.86	31	0	2.23	0.66
freq ITWAC lemma	31	0	1.39	0.68	31	0	2.39	0.63
	Noun				Verb			
	valid	*miss %*	*mean*	*st.dev.*	*valid*	*Miss %*	*mean*	*st.dev.*
freq. Repubblica form	31	0	3.90	0.76	18	41.94	3.73	0.9
freq. ITWAC form	31	0	3.78	0.57	23	25.81	4.07	1.16
freq. Repubblica lemma	31	0	3.67	1.09	31	0	3.80	0.71
freq. ITWAC lemma	31	0	3.90	0.72	31	0	3.72	0.71

Ext. Data Table 2: Surprisal values analyses. Number of valid cases, percentage of missing, mean and the standard deviation relative to the surprisal value, separately for the two experimental conditions (Noun-phrase, Verb-phrase).

	Low Surprisal	High Surprisal	tot (n)
noun-phrase	26	5	31
verb-phrase	5	26	31
tot (n)	31	31	

Ext Data Table 3: distribution of the surprisal values of articles and clitics on the basis of the median (M = 1.9097) obtained from the occurrence of the lemma in the ITWAC database.

Number of patients	10
Gender	5 male and 5 female
Median age at SEEG	32 (range 17-44)
Median years of scholarization	13 (range 0-17)
Median age of onset of epilepsy	16 (range 2-30)
Median duration of epilepsy (years)	15 (range 3-30)

Ext. Data Table 4: Demographic data. Summary of demographic data of patients that successfully completed the study. SEEG: Stereo-Electro-Encephalography.
